# A Novel Dual-Gradient Patterned Wettability Current Collector for Passive DMFCs

**DOI:** 10.3390/nano16090518

**Published:** 2026-04-25

**Authors:** Yingli Zhu, Leyao Ban, Yingying Jing, Yangyang Cheng

**Affiliations:** School of Mechanical Engineering, Tianjin University of Science and Technology, Tianjin 300457, China; 15332199000@163.com (L.B.); 16634864090@163.com (Y.J.); 19966126335@163.com (Y.C.)

**Keywords:** direct methanol fuel cells, current collector, dual-gradient wettability, gas–liquid management, mass transfer

## Abstract

Direct methanol fuel cells (DMFCs) offer significant advantages including high energy density and rapid refueling, making them promising power sources for portable electronic products. However, their practical application, particularly in passive systems, is hindered by critical mass transport limitations: water flooding in the cathode and CO_2_ bubble blockage in the anode. Herein, a novel dual-gradient patterned wettability current collector (CC) was designed to alleviate this mass transport impedance. The design uniquely integrates wedge-shaped gradients with surface energy gradients to create a unified, self-driven mechanism for efficient water and CO_2_ bubble transport at both electrodes. A mathematical model was developed to quantitatively evaluate the effects of the dual-gradient structure. The results confirm that water removal is enhanced when the cathode current collector features a hydrophobic periphery with a dual-gradient patterned wettability interior on the gas-diffusion-layer side and a fully hydrophilic air-side surface, whereas an inverted pattern facilitates anode CO_2_ removal. Optimal fabrication parameters on 316 L stainless steel were established by investigating laser scanning conditions and low-surface-energy agent concentrations. The experimental results show that the passive DMFCs incorporating the optimized current collectors delivered marked performance improvements. At 1 mol·L^−1^ methanol, the novel anode and cathode current collectors increased peak power density by 15.6% and 14.5%, respectively. Electrochemical impedance spectroscopy revealed a 31.4% and 31.9% reduction in mass transfer resistance of the cell with novel anode and cathode current collectors, respectively, confirming improved gas–liquid self-driven efficiency. Furthermore, the new cells exhibited substantially enhanced long-term stability over 18 h of continuous discharge, attributed to the robust wettability achieved via laser–silane modification. Overall, these findings suggest that the proposed dual-gradient wettability design is a promising method for improving internal mass transport, potentially supporting the development of more robust passive DMFCs.

## 1. Introduction

Although direct methanol fuel cells (DMFCs) offer a theoretical energy density of up to 6000 Wh·kg^−1^—five times higher than lithium-ion batteries—their practical energy density is limited to approximately 500 Wh·kg^−1^ by challenges including low catalyst efficiency, methanol crossover, and complex hydrothermal management; however, it is still 2 times that of lithium-ion batteries [1]. Therefore, DMFCs are regarded as promising power sources for portable electronic devices. However, DMFCs still encounter technical challenges hindering large-scale commercialization, including water flooding [2] in the cathode and carbon dioxide blockage [3] in the anode.

During DMFC operation, carbon dioxide (CO_2_)—a primary anodic reaction product—accumulates continuously. Failure to release CO_2_ promptly induces gas blockage, which increases methanol mass transfer resistance and consequently degrades cell performance [4,5]. To address this issue, many scholars have attempted to remove CO_2_ bubbles efficiently to alleviate mass transfer blockage in two-phase flows [6,7]. Some researchers have focused on modifying the microstructure of the current collector (CC) to achieve better anode CO_2_ management. For instance, Su et al. [8] introduced a sinusoidal corrugated channel into single-serpentine flow fields to enhance the fluid flow rate within the flow channel, thereby facilitating bubble detachment from the anode side. Yuan et al. [9] visually investigated bubble behavior under three flow field designs—namely, the traditional right-angle serpentine, the rounded-angled serpentine, and the stepwise broadening serpentine flow fields—and found that CO_2_ bubble motion was closely correlated with the pressure drop in the flow field. However, another group of scholars has focused on promoting the expulsion of anodic CO_2_ bubbles by modifying the wettability of the internal cell components. Osman et al. [10] incorporated a hydrophobic degassing channel between the anode flow field and the anode end plate to drive bubbles out of the flow channel, thereby eliminating channel blockage. To reduce the methanol transport resistance, Li et al. [11] proposed fabricating a series of superhydrophobic lateral venting microchannels on the surface of the anode endplate around the gas diffusion electrode, allowing CO_2_ bubbles to be released directly from the gas diffusion layer (GDL).

An electrochemical reaction at the cathode produces water during DMFC operation. Additionally, water from the anode migrates to the cathode via diffusion, electro-osmosis, and methanol crossover, further increasing the cathode water content. If this water is not discharged in a timely manner, flooding occurs, blocking oxygen mass transfer pathways and inducing concentration polarization, which ultimately degrades cell performance. To address this issue, Xue et al. [12] developed a novel cathode gas diffusion layer composed of reduced graphene oxide and stainless steel fiber felt, leveraging the hydrophilic and dispersive properties of the composite to capture and rapidly disperse water, thereby mitigating flooding. Su et al. [13] constructed hydrophilic stepped channels on the cathode current collector, utilizing capillary forces to drive water toward evaporation zones where it is rapidly evaporated into the ambient air. Shu et al. [14] fabricated a novel GDL by combining vacuum-filtered carbon fibers with multi-walled carbon nanotubes, creating a multi-stage pore structure that facilitates water transport and uniform gas distribution. Zhang et al. [15] evaluated the performance of a µDMFC equipped with a cathode current collector assembly featuring gradient wettability, and found that the gradient force generated by such a collector promotes water discharge.

In this study, a conventional current collector was modified by constructing a dual-gradient patterned wettability structure on its surface. Although numerous studies have explored strategies to mitigate either cathode flooding or anode CO_2_ blockage through macrostructure modification or localized wettability alteration, a unified approach capable of simultaneously addressing both mass transport challenges across the two electrodes remains largely unexplored. Existing methods typically focus on single-electrode modifications or rely on single-gradient designs, offering only partial solutions. Herein, we introduce a novel dual-gradient patterned wettability current collector designed to overcome these limitations. The core novelty lies in the integration of precisely engineered wedge-shaped l gradients with surface energy gradients on the current collector surface, creating a unique, self-driven mechanism that promotes efficient and directional transport of water at the cathode and CO_2_ bubbles at the anode. This integrated design represents a significant advancement over conventional or single-gradient approaches. The performance of the novel current collector bearing the dual-gradient patterned wettability structure was compared with that of a conventional current collector through simulations and experimental tests, focusing on CO_2_ bubble release characteristics and flooding mitigation capability.

## 2. Simulation

### 2.1. Design of Dual-Gradient Patterned Wettability Current Collector

**Figure 1:**
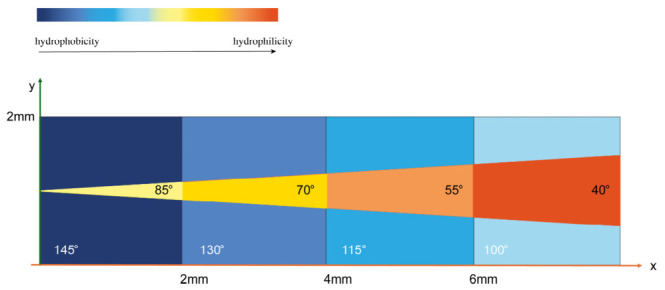
Hydrophilic wedge arrays fabricated on gradient hydrophobic surfaces.

To enhance transport efficiency, wedge-shaped patterns were fabricated on a gradient hydrophobic substrate, as shown in Figure 1, with the horizontal region divided into hydrophobic (blue zone, step contact angles: 145°, 130°, 115°, 100°) and hydrophilic (orange zone, step contact angles: 85°, 70°, 55°, 40°) zones. Droplets and bubbles acquire an initial velocity from the Laplace pressure at the tip. As they traverse the hydrophobic–hydrophilic steps simultaneously, they are continuously accelerated by an augmented gradient force arising from surface tension imbalance, thereby promoting their expulsion.

**Figure 2:**
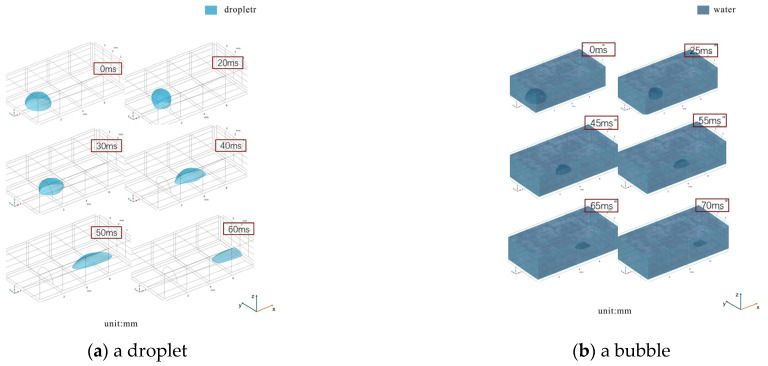
Migration over wedge-shaped dual wettability gradients. (**a**) droplet; (**b**) bubble.

A wedge angle of 9° and a pattern length of 4 mm were selected for practical relevance, with the simulation model shown in Figure 2. The laminar two-phase flow phase field multiphysics interface was employed, using an average mesh size of 0.05 mm, which was validated via mesh independence tests to balance computational efficiency and reliability.

For gas self-migration simulations, an air–water incompressible laminar flow system was adopted, with phase-field variables defined as 1 (liquid) and −1 (gas, primary phase). A hemispherical bubble was initially positioned at the wedge tip, with the bottom boundary set as wettability-controlled with gradient contact angles, and the right boundary as the outlet.

As shown in Figure 2a, a droplet spontaneously migrates toward the wider end of the wedge. It remains stationary at t = 0 ms, exhibits a flattened front and convex rear at 30 ms, and coalesces and is expelled by 50 ms, demonstrating a higher migration rate than that on purely gradient surfaces. Figure 2b illustrates bubble transport. The bubble is stationary at t = 0 ms, then driven by the wedge-induced Laplace pressure difference to advance, showing a flattened front at 45 ms, and exits through the outlet by 55 ms.

To minimize contact resistance between the current collector and the gas diffusion layer, an open area ratio of 30–60% was adopted to balance conductivity and mechanical strength. The super hydrophobic cathode current collector (30 mm × 30 mm, with an active area of 16 mm × 16 mm) features a hole pitch of 3 mm and a hole depth of 0.5 mm, and drainage holes 0.5 mm in diameter, resulting in an open area ratio of 44.2%.

### 2.2. Verification and Validation of the Model

Based on the three-dimensional modeling of the DMFC, this section further investigates the impact of the novel current collector on DMFC performance. Leveraging the symmetric characteristics of the DMFC structure, the computational domain is simplified to one-fourth of the actual model, thereby reducing computation time while maintaining computational efficiency. Only the 2 mm × 2 mm through-hole area is selected as the object of flow field analysis. The concentration of methanol solution is set to 1 mol·L^−1^, and the temperature is maintained at 60 °C. All other environmental parameters and configurations remain the same as those described above. The specific physical characteristics of the gas and liquid phases are shown in Table 1.

**Figure 3:**
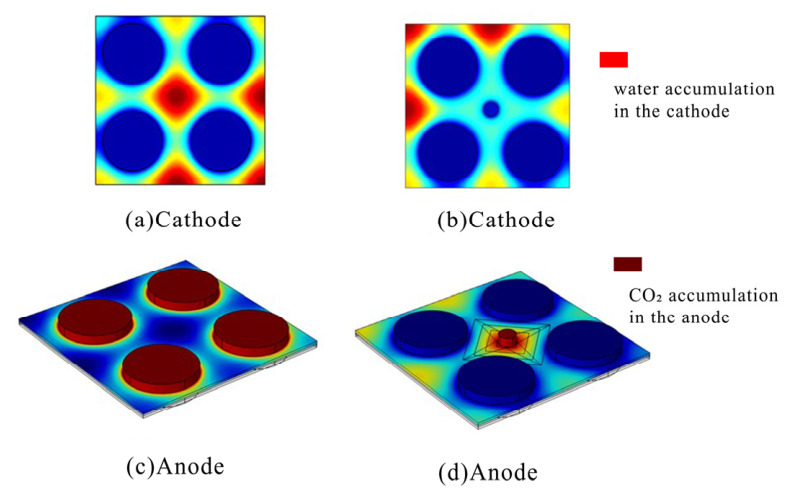
(**a**) Water accumulation in the DMFC with the conventional cathode CC. (**b**) Water accumulation in the DMFC with the modified cathode CC. (**c**) CO_2_ accumulation in the DMFC with the conventional anode CC. (**d**) CO_2_ accumulation in the DMFC with the modified anode CC.

The simulation results demonstrate that severe cathode flooding occurs in the absence of the novel cathode current collector (Figure 3a). With the integration of this collector, droplets are either expelled through the perforated holes or directed toward the wider end via the synergistic effect of the dual-gradient wedge-induced Laplace force and wettability gradient force, enabling efficient gas–liquid separation (Figure 3b). On the anode side, bubbles slide into the wedge-shaped structures immediately upon making contact with the patterned surface. Under the co-directional coupling of the dual-gradient wedge-induced Laplace force and wettability gradient force, the bubbles are propelled toward the wider end and vented through the exhaust ports. Subsequently, the bubbles escape through the hydrophobic back-side region with the assistance of buoyancy, ensuring that the fuel inlet remains in a liquid phase without blockage (Figure 3d). These results confirm that the dual-gradient structure mitigates both cathode flooding and anode CO_2_ accumulation simultaneously.

**Figure 4:**
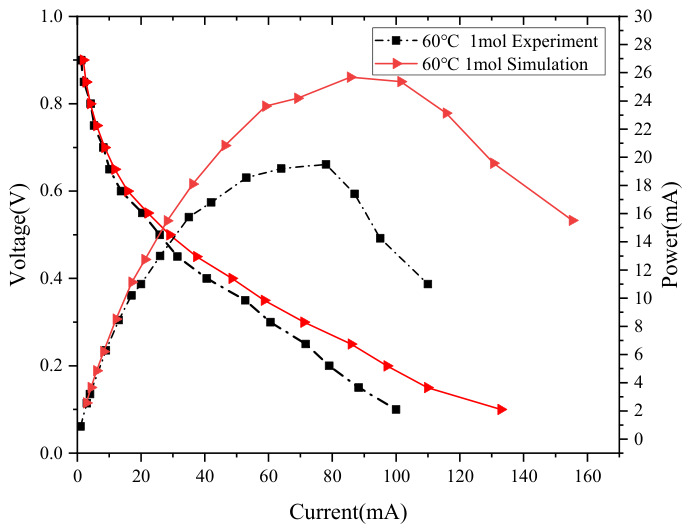
Comparison of simulation results and experimental results.

The performance of the DMFC was investigated at various methanol concentrations and temperatures of 40 °C, 50 °C, and 60 °C, and the corresponding polarization curves were obtained. The simulated polarization curves were compared with the experimental data, as presented in Figure 4 and Table 2. The simulated curves are in good agreement with the experimental ones in terms of the overall trend. Under methanol concentrations of 1–2 mol·L^−1^ and temperatures of 40–60 °C, both curves exhibit typical behavior where the voltage decreases gradually with increasing current density, confirming that the established model can accurately capture the operating characteristics of the DMFC under different working conditions. In the low-current-density region, the simulation and experimental results match well, indicating that the model can precisely describe the electrochemical reaction kinetics that dominate cell performance at low loads. As the current density increases, slight deviations can be observed at some points, but the overall variation tendency remains consistent.

## 3. Experimental

### 3.1. Wettability Treatment Process for Current Collector 

**Figure 5:**
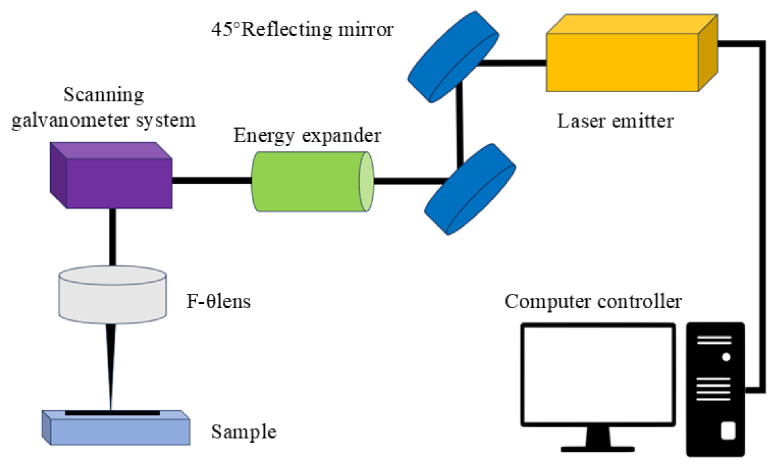
Schematic diagram of laser processing device.

316L stainless steel was processed into 30 mm × 30 mm × 0.4 mm square thin sheets as experimental substrates. Prior to laser processing, samples were ultrasonically cleaned in anhydrous ethanol for 10 min, rinsed with deionized water, and dried with nitrogen to ensure surface cleanliness. Laser etching was conducted using a Han’s Laser EP-20-SHG (Han’s Laser Technology Industry Group Co., Ltd., Shenzhen, China) equipment. The laser beam, guided by two 45° reflecting mirrors into a scanning galvanometer and focused to approximately 20 μm via an F-θ lens, carried out high-precision local ablation on the stainless steel surface according to the preset path shown in Figure 5. Key processing parameters included a power of 10 W, a wavelength of 532 nm, a focal length of 224 mm, and a pulse frequency of 10–200 kHz, resulting in significant changes in surface microstructure and increased roughness.

Laser scanning was performed at speeds of 0.4–3 m·s^−1^ and spacings of 15–45 μm. Post-processing static contact angle measurements were conducted to assess the effects of these parameters on surface wettability. After laser etching, samples were immersed in a 1–5% ethanol solution of triethoxysilane, then retrieved for heat curing, and characterized for wettability using a contact angle meter. Optimal laser-chemical modification parameters were selected to fabricate super hydrophobic stainless-steel surfaces, with the experimental procedure shown in Figure 5.

**Figure 6:**
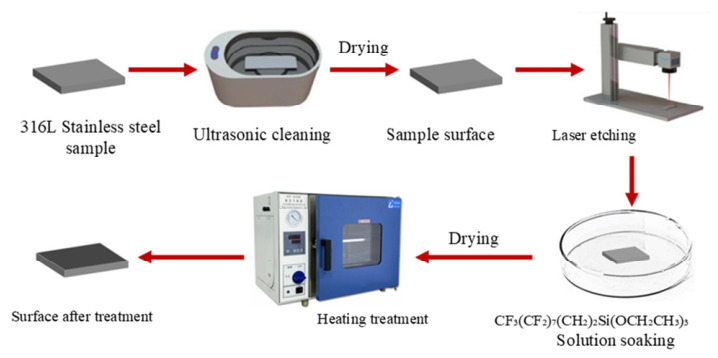
Schematic diagram of wettability treatment process.

As shown in Figure 6, laser etching enabled high-precision tailoring of surface hydrophobicity by adjusting the scanning spacing (3–50 μm) and speed (0.6–10 m·s^−1^) for contact angle fine-tuning. Static contact angles and roll-off angles were measured five times at five random locations using 2 μL deionized water droplets (via five-point fitting, height, and angle methods) to ensure statistical reliability. A quantitative model correlating process parameters with surface properties was established, and curve fitting for gradient hydrophobic requirements yielded optimal laser speed–spacing combinations for target contact angles.

To simulate real DMFC operating conditions, 316L samples with fixed laser parameters and silane post-treatment underwent a 30-day stability test. The air-exposed group showed a slight reduction in contact angle attributed to dust deposition but retained high hydrophobicity. The water-immersion group, with air isolation and minimal organic contamination, exhibited a slower decline, maintaining superhydrophobicity after 30 days. These results confirm the excellent durability of the micro-/nano-composite structure in both gaseous and liquid environments.

### 3.2. Materials and Experimental Platform for DMFC

**Figure 7:**
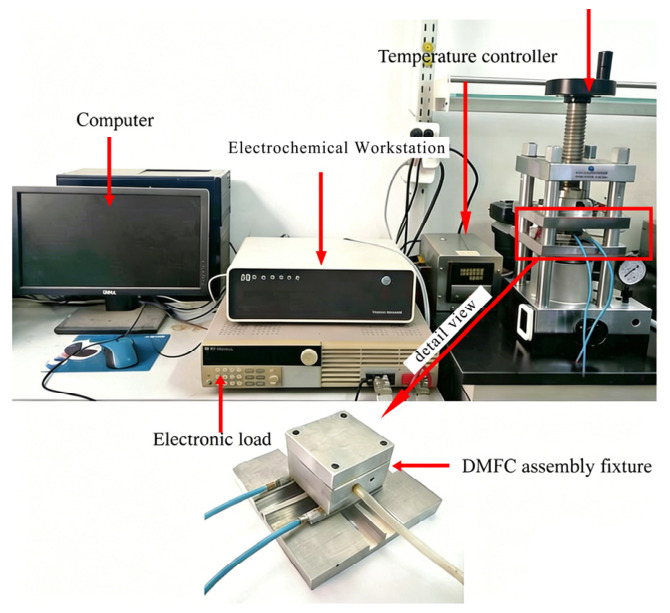
The experimental platform of DMFC.

The materials and experimental platform used for the DMFC are shown in Table 3 and Figure 7, respectively. This study focused on a horizontally placed single DMFC. Methanol solution was injected into the anode chamber using a microsyringe, with the exhaust hole located on the sidewall. The cathode operated in an air-breathing mode, obtaining oxygen from ambient air.

In this experiment, an ITECH 8500 electronic load [M18.1] (ITECH Electronics Co., Ltd., Nanjing, China) and a CHI660D electrochemical workstation (Shanghai Chenhua Instrument Co., Ltd., Shanghai, China) were used to measure the polarization curve and the electrochemical impedance spectroscopy (EIS) of the DMFC to evaluate its performance. To eliminate interference from temperature and pressure fluctuations and ensure reproducible testing, a temperature-controlled fuel cell fixture was used to maintain stable operating conditions.

From both the simulation and experimental results, it is observed that when the methanol concentration exceeds 2 mol·L^−1^, the fuel cell performance declines rapidly due to severe methanol crossover. Therefore, the methanol concentration in the subsequent experiments was set in the range of 0.5–2 mol·L^−1^.

## 4. Results and Discussion

### 4.1. New Current Collector and Its Performance

**Figure 8:**
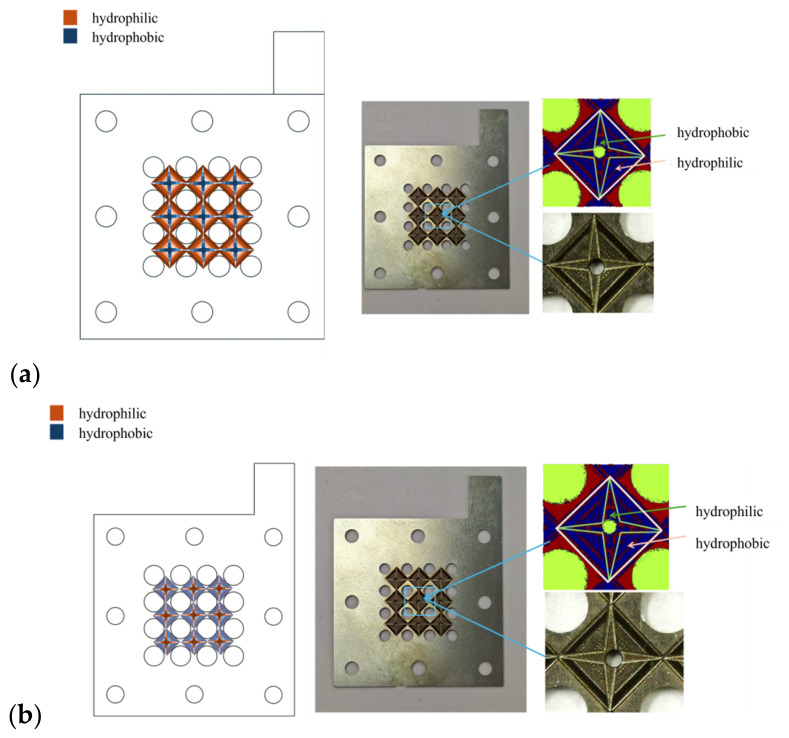
Schematic diagram of surface treatment of new CC. (**a**) Anode; (**b**) cathode.

As shown in Figure 8, a dual-gradient patterned wettability structure was fabricated on the side of the anode current collector that is in direct contact with the gas diffusion layer, while the back side of the anode current collector is rendered hydrophobic. By modifying the surface wettability of the anode current collector, CO_2_ bubbles can be rapidly expelled from the anode flow field, thereby reducing bubble accumulation. The cathode current collector features the same microstructure as the anode current collector, but with the opposite wettability pattern.

To verify the enhancement effect of the anode dual-gradient patterned wettability structure, a conventional dot-type cathode current collector was employed as a unified control, and the reference cell was compared with the anode-improved cell. Conversely, when testing the novel cathode current collector, a conventional dot-type anode current collector was employed for the DMFC.

**Figure 9:**
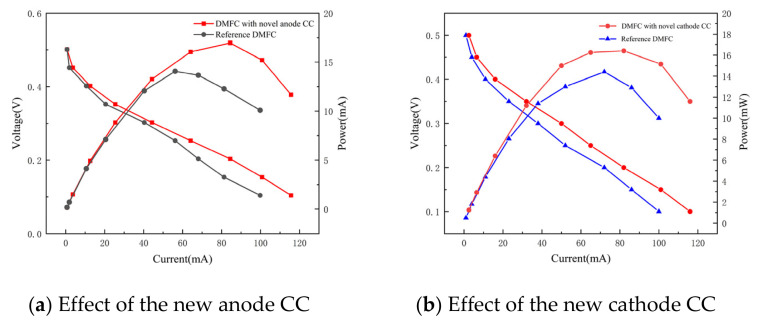
Output performance of the DMFC. (**a**) Effect of the new anode CC; (**b**) Effect of the new cathode CC.

Figure 9a presents the polarization and power density curves of the DMFC with the novel anode CC at a methanol concentration of 1 mol·L^−1^. In the low-current region, the two cells exhibit nearly identical performance, whereas in the high-current region, the improved anode cell reaches a higher peak power first. The peak power density of the improved anode cell increases by 15.67%, confirming that the dual-gradient structure can expel the generated CO_2_, alleviate inlet blockage, and thereby enhance output performance.

Figure 9b shows the measured voltage–current characteristic curves and power density curves of the DMFC with the novel cathode CC. The experimental results indicate that when the cell operating voltage is 0.2 V, the cell reaches its peak power. Under the conditions of 40 °C and 1 mol·L^−1^ methanol concentration, the peak power of the reference cell is 14.4 mW, while the cell with the improved cathode current collector reaches a peak power of 16.5 mW, corresponding to an overall power increase of 14.5%.

### 4.2. Fitting of the Equivalent Circuit for the DMFC

**Figure 10:**
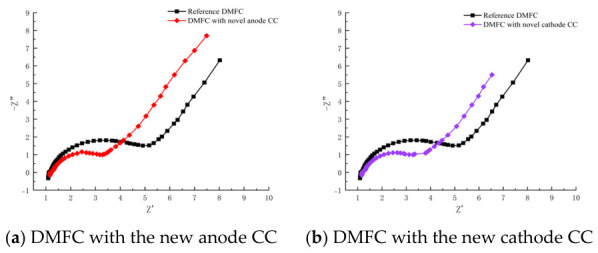
Nyquist plots of the DMFC. (**a**) DMFC with the new anode CC; (**b**) DMFC with the new cathode CC.

**Figure 11:**
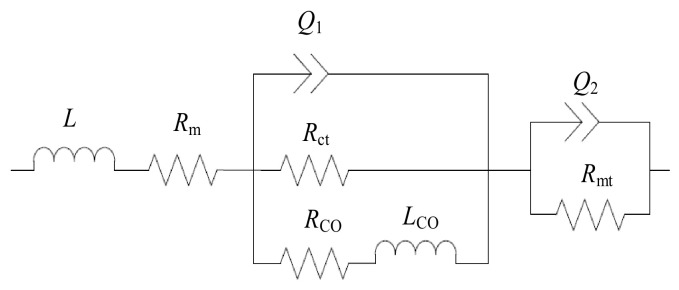
Equivalent circuit of the DMFC.

Electrochemical impedance spectroscopy was employed as a supplementary evaluation index to investigate cell electrochemical reactions, particularly for charge transfer- and diffusion-controlled electrode processes. Figure 10 presents Nyquist plots of the DMFC under 50 °C and 1 mol·L^−1^ methanol. The equivalent circuit method [16] is often used to evaluate the various impedances of a fuel cell. Herein, as shown in Figure 11, the Nyquist plots were fitted using an equivalent circuit model consisting of ohmic resistance (R_m_), charge transfer resistance (R_ct_), mass transfer resistance (R_mt_), and a constant phase element (CPE, Q_1_ and Q_2_ in Figure 11). The resistance R_CO_ and the low-frequency impedance L_CO_ reflect the complex relaxation process of the anodic intermediate product CO.

The fitted parameters are summarized in Table 4. Under the conditions of 50 °C and 1 mol·L^−1^ methanol, the DMFC with the novel anode CC exhibits a mass transfer resistance of 2.612 Ω, which is 31.4% lower than that of the reference DMFC (3.806 Ω). This result verifies that the dual-gradient wettability structure effectively facilitates the removal of product water and CO_2_ bubbles, thereby significantly alleviating mass transfer polarization. The charge transfer resistance of the cell with the novel anode CC is 1.068 Ω, comparable to 1.006 Ω for the reference cell, suggesting that the surface modification imposes no adverse effect on the electrochemical reaction kinetics. The ohmic resistance increases slightly to 1.162 Ω due to laser etching and silane modification; however, this minor increment is fully compensated by the remarkable reduction in mass transfer resistance.

The DMFC with the novel cathode current collector also exhibits superior electrochemical impedance characteristics, with a mass transfer resistance of 2.591 Ω, 31.9% lower than that of the reference DMFC. This further confirms the validity of the wettability gradient design in accelerating water removal and mitigating concentration polarization. The charge transfer resistance and ohmic resistance of the novel cathode cell are 1.087 Ω and 1.131 Ω, respectively, both close to those of the reference cell.

**Figure 12:**
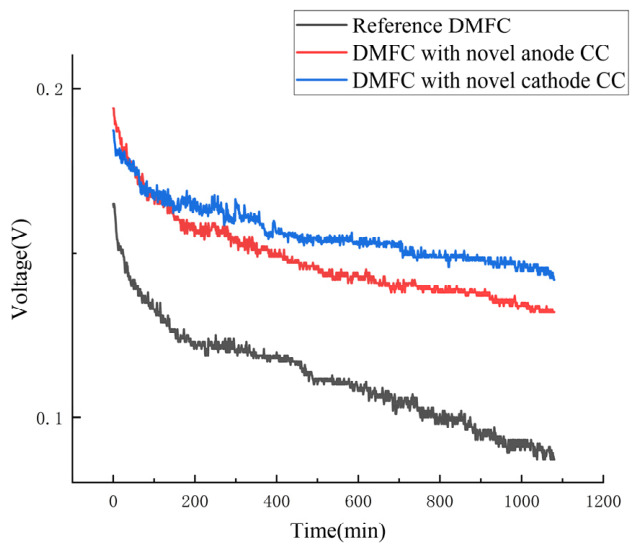
Long-term constant-current discharge performance.

Long-term discharge tests were performed on the modified and reference cells under a constant current of 90 mA for 18 h with continuous methanol supply, as shown in Figure 12. The reference cell showed significant performance degradation, while the novel cell exhibited minimal decline. These results further demonstrate that, driven by the Laplace gradient force and the wettability gradient force, the novel current collector enables self-transport of CO_2_ and water droplets through the synergy between the gradient wettability and the wedge-shaped pattern surfaces. Consequently, it effectively guides and expels liquid water and CO_2_, maintaining flow field patency and oxygen transport efficiency, thereby enhancing cell lifespan and long-term stability.

## 5. Conclusions

This study introduced a dual-gradient patterned wettability current collector to address the critical issues of cathode flooding and anode CO_2_ blockage in passive DMFCs. The main findings are as follows. First, the integration of wedge-shaped topological gradients with surface energy gradients creates a self-driven transport mechanism, enabling directional water removal at the cathode and rapid CO_2_ bubble detachment at the anode. Second, systematic optimization of laser processing parameters on 316L stainless steel yielded a stable superhydrophobic surface that maintained its wettability over 18 h of continuous operation. Third, under identical operating conditions (1 mol·L^−1^ methanol, 60 °C), the novel anode and cathode current collectors increased peak power by 15.6% and 14.5%, while reducing mass transfer resistance by 31.4% and 31.9%, respectively, as confirmed by EIS.

Compared with previous studies that typically focused on single-electrode modifications (e.g., anode flow field redesign [8,9,10] or cathode GDL modification [12,13,14,15]), the present work offers a unified solution that simultaneously improves gas–liquid management at both electrodes. The dual-gradient design achieves comparable or better performance gains without requiring complex external auxiliary components.

Despite these advantages, several limitations remain. The optimal matching relationship between wedge angle and wettability gradient has not yet been systematically investigated, and orthogonal experiments are needed to further enhance performance.

## Figures and Tables

**Table 1:** Physical properties of gas phase and liquid phase.

Parameters	Unit	Value
Water density	kg·m^−3^	1000
Air density	kg·m^−3^	1.29
Water dynamic viscosity	Pa·s	1.01 × 10^−3^
Air dynamic viscosity	Pa·s	1.8 × 10^−5^
Surface tension coefficient	N·m^−1^	0.072

**Table 2:** Comparison of simulated and experimental DMFC performance.

Experimental Condition	Peak Power of Simulation (mW)	Peak Power of Experiment (mW)
60 °C, 1 mol·L^−1^	25.4	19.5
50 °C, 1 mol·L^−1^	21.5	18.1
40 °C, 1 mol·L^−1^	18.2	14.4
60 °C, 2 mol·L^−1^	24.5	17.4
50 °C, 2 mol·L^−1^	21.2	14.5
40 °C, 2 mol·L^−1^	17.7	13.4

**Table 3:** Materials used in the research of this paper.

Material	Specification	Supplier
Membrane	Nafion, 183 μm	DuPont, Wilmington, DE, USA
Anode catalyst	Pt, Ru, C	Shanghai Hesen Electric Shanghai, China
Cathode catalyst	Pt, C	Shanghai Hesen Electric, Shanghai, China
Carbon paper	TGP-090, 0.28 mm	Toray, Tokyo, Japan
Membrane electrode assembly (MEA)	Active area: 16 mm × 16 mm	Toray, Tokyo, Japan
Methanol	CH_3_OH	Sinopharm Chemical Reagent Co., Ltd., Shanghai, China
Current collector	316L stainless steel, 0.4 mm	Shenzhen Yaoda Precision, Shenzhen, Guangdong, China
Gasket	xSiO_2_·yH_2_O	Shenzhen Huaren Plastic, Shenzhen, Guangdong, China

**Table 4:** EIS fitting parameters.

Cell Type	R_m_ (Ω)	R_ct_ (Ω)	R_mt_ (Ω)
Reference cell	1.094	1.006	3.806
DMFC with novel anode CC	1.162	1.068	2.612
DMFC with novel cathode CC	1.131	1.087	2.591

## Data Availability

The original contributions presented in this study are included in the article. Further inquiries can be directed to the corresponding author.
